# Secondary Acquired Cholesteatoma in Langerhans Cell Histiocytosis Patient

**DOI:** 10.7759/cureus.32173

**Published:** 2022-12-04

**Authors:** Shaden Alhelali, Nawaf Alsulami, Linah Qasim, Kamal Hanbazazah, Yahya Khubrani, Rehab Fadag

**Affiliations:** 1 Department of Otorhinolaryngology Head and Neck Surgery, King Fahad Armed Forces Hospital, Jeddah, SAU; 2 Department of Otolaryngology Head and Neck Surgery, King Fahad Armed Forces Hospital, Jeddah, SAU; 3 Department of Otolaryngology Head and Neck Surgery, King Faisal Specialist Hospital and Research Center, Jeddah, SAU; 4 Department of Histopathology, King Fahad Armed Forces Hospital, Jeddah, SAU

**Keywords:** temporal bone langerhans cell histiocytosis, mastoid, langerhans cell histiocytosis, acquired cholesteatoma, cholesteatoma

## Abstract

Langerhans cell histiocytosis (LCH) is a multi-faceted disease defined by the accumulation of dendritic cells in various organs with characteristics similar to the epidermal Langerhans cells and can affect any organ of the body. It is most commonly seen in young adults and children. Cholesteatoma is a congenital or acquired condition and is categorized into primary and secondary cholesteatomas. Only a few reported cases of primary or secondary cholesteatoma have been reported among patients treated for temporal bone LCH. We report a case of secondary acquired cholesteatoma in a six-year-old girl after five years of her LCH treatment. The patient initially presented with ear discharge and aural polyp which did not improve with medical management. A computed tomography scan of the mastoid showed a left middle ear cavity and temporal bone lesion with bony erosions and total obliteration of the left external auditory canal. The patient underwent multiple biopsies, and a histopathological evaluation confirmed the diagnosis of cholesteatoma. The diagnosis of LCH can be difficult due to variable clinical manifestations. Involvement of the ear as resemblance to other diseases such as mastoiditis and chronic otitis media are quite common. Computed tomography scan and biopsy are reliable tools for diagnosis. Cholesteatoma following LCH remains a rare entity, hence, critical examination at follow-up visits is needed. Surgery remains the treatment of choice for cholesteatoma patients.

## Introduction

Langerhans cell histiocytosis (LCH) is a heterogeneous disease defined by an accumulation of dendritic cells in diverse organs with characteristics comparable to epidermal Langerhans cells. Moreover, the skeleton (80%), skin (33%), and pituitary (25%) are the most affected sites by the disease. Other organs that are affected include the liver, spleen, hematological system, lungs, lymph nodes, and the central nervous system. The disease's clinical course can range from self-limiting to rapidly progressing and potentially fatal. Approximately, around 30% and 40% of the patients may experience long-term complications [1]. It is more common among young adults and children. In children, its prevalence rate can reach up to three to five children per million. The head and neck areas account for about 50% to 80% of pediatric LCH. In 15% to 60% of this location, the temporal bone is affected. Mastoid swelling or temporal bone mass, otalgia, and otorrhea are all otologic symptoms. Because the otologic findings are so similar to those of otitis media, otitis externa, cholesteatoma, and other disorders, diagnosis is often delayed [2].

Approximately 15% of patients with LCH have auricular involvement and clinical manifestations are not always clear. Retraction pockets in the pars tensa and flaccida cause acquired cholesteatoma. On the other hand, secondary acquired cholesteatoma develops as a result of tympanic perforation or skin entrapment which is frequently reported after an ear injury or surgery. Cholesteatoma formation in LCH patients is an uncommon occurrence. Cholesteatoma formation appears to occur at either the external auditory canal or mastoid in about 2-3 years after a patient is diagnosed with LCH [3]. Patients with LCH are no more likely to be diagnosed with cholesteatoma in comparison to the general population. They are more likely to be diagnosed with chronic otitis externa, hence, it should be distinguished from cholesteatoma or recurrence of LCH [4].

LCH is diagnosed based on clinical signs and symptoms, radiographic findings, and histological findings. Positive CD1a and/or S100 antigen are immunohistochemical markers of LCH. The major treatment options are surgery and radiation. In individuals with aggressive, disseminated and resistant lesions, medical treatment should be administered. The prognosis of the disease is determined by age and the extent of organ involvement [5]. We report a case of a 6-year-old girl who was diagnosed with cholesteatoma five years after her treatment of LCH of the temporal bone.

## Case presentation

**Figure 1:**
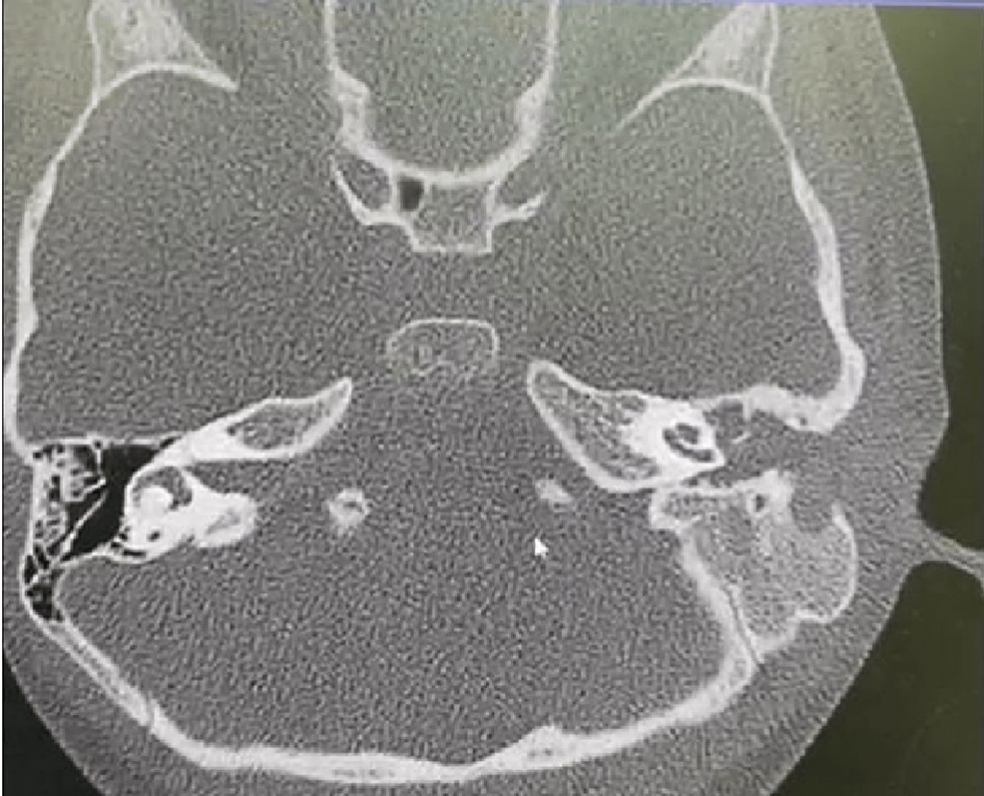
Non-enhanced temporal bone computed tomography scan showing soft tissue lesion totally obliterating the left middle ear cavity and left mastoid air cells, engulfing its bony ossicles

We report a case of a 6-year-old girl who was diagnosed with LCH of the left temporal bone and was treated successfully with surgery at the age of one year. The surgical biopsy confirmed her diagnosis of LCH, and she was placed on chemotherapy. She had regular follow-up appointments at intervals of three months at the paediatric oncology and otolaryngology departments. During her follow-up visits, there was no complaint, however, in March 2021, she presented with ear discharge and aural polyp. She was then managed with medicines but showed no signs of improvement. The young patient was then referred for a Computed Tomography (CT) scan of the temporal bone. A non-enhanced temporal bone CT scan showed a soft tissue lesion totally obliterating the left middle ear cavity and left mastoid air cells. It was engulfing the bony ossicles and the soft tissue density/lesion was obliterating the left external auditory canal. In addition, bony erosions of the temporal/mastoid bone along with the erosion of the scutum were found (Figure 1). 

**Figure 2:**
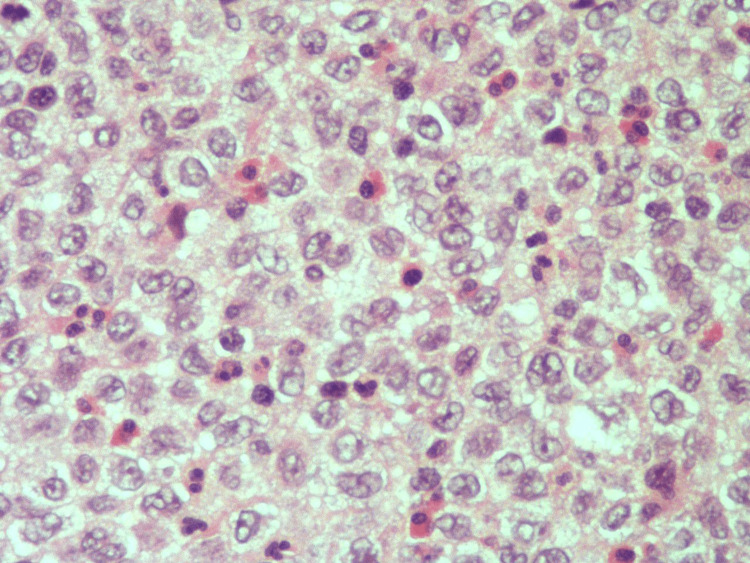
Oval mononuclear cells with reniform and cleaved nuclei with pale cytoplasm. Interspersed eosinophils were also present (hematoxylin-eosin, original magnification x40)

**Figure 3:**
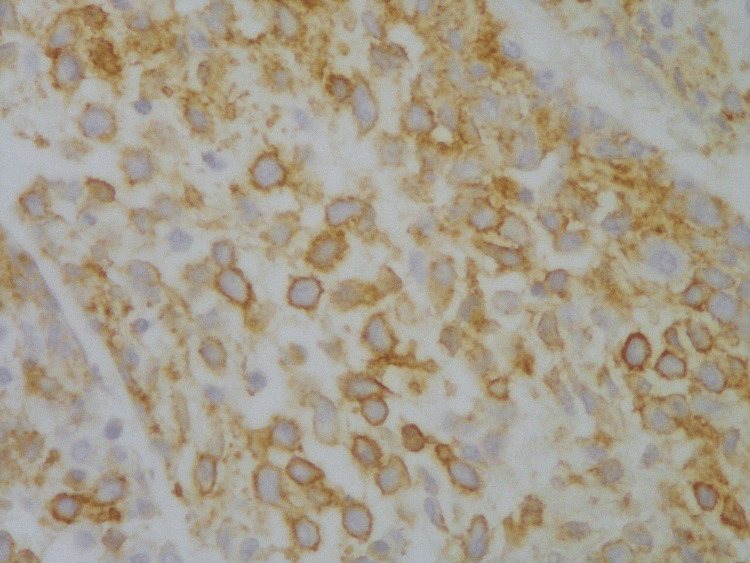
CD1 immunohistopathology slide showing positive staining in the tumor cell (original magnification x40)

**Figure 4:**
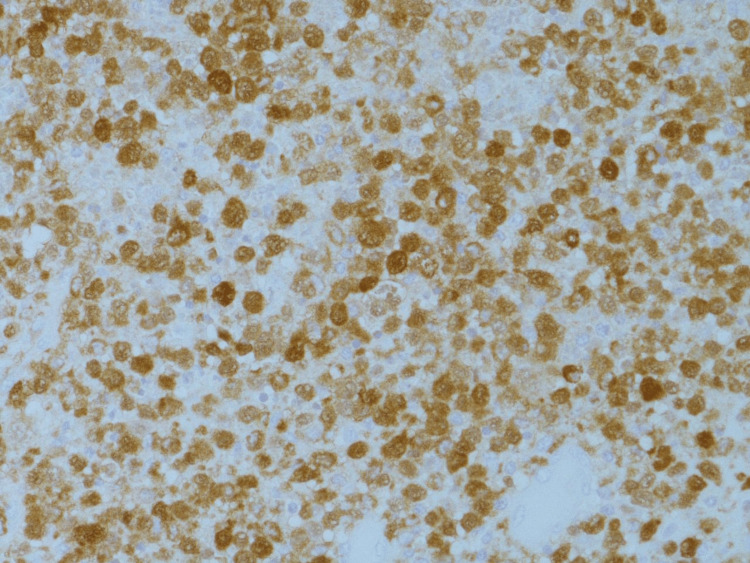
S100 immunohistopathology slide showing positive staining in tumor cells (original magnification x40)

A multidisciplinary team reviewed the case and concluded to plan for a biopsy of the newly developed lesion to determine the further course of action for effective management of the patient. The young girl was then taken to the operation theatre, where she underwent canal wall down mastoidectomy and multiple biopsies of the mastoid, middle ear and external ear lesions. The previous tumor of Langerhans cell histiocytosis was diagnosed in 2015 and shows sheets of oval mononuclear cells with reniform and cleaved nuclei with pale cytoplasm. Interspersed eosinophils were also present (Figure 2). The histopathological findings were positive for CD68 in the lung biopsy and negative for CD1 and S100 (Figures 3, 4). The diagnosis of cholesteatoma was confirmed with no reported recurrence.

## Discussion

LCH refers to a collection of disorders caused by the clonal expansion of Langerhans cells. The etiopathogenesis of the disease is yet unknown, with a rare occurrence. The prevalence is roughly 5.4 per million, with a peak in children aged 1 to 4. However, it can affect people of any age. Many authors have speculated about immunological, genetic, or viral predisposing factors, but no clear evidence has been found. There are no pathognomonic characteristics, and the clinical manifestation differs from patient to patient. The involvement of the external and middle ear is found often, but it is frequently misdiagnosed due to the otologic findings. Similarities to those of other conditions such as mastoiditis, chronic otitis media, recurrent otitis externa, and stenosis can make the diagnosis difficult [6].

Ali and Al-Kindy reported a case of a 22-month-old Saudi boy who had severe mastoiditis with a fistula that refused to respond to traditional treatment. A lytic lesion of the temporal bone was discovered on a CT scan. The cavity of the mastoid was full of friable and gelatinous tissue, which raised suspicions of LCH, and was later validated by histopathological and immunohistochemical tests CD1a and S100 [7]. Immunohistochemistry of our patient revealed a positive CD68 but was negative for both CD1a and S100. Sayore et al. reported a case of a 4-year-old patient with otorrhea and recurrent otalgia who developed left axillary non-blowing exophthalmia over the course of 5 months. The authors stated that it was accompanied by indurated left temporal swelling and indurated swelling of the posterior face of the left shoulder due to a pathological fracture. An extra-conical process with extension at the temporal fossa was discovered on cerebro-orbital magnetic resonance imaging. After biopsy of the temporal tumour, the pathological examination revealed that the patient had LCH. Immunohistochemical results of the S-100 protein and the CD1 antigen were used to make the diagnosis. Chemotherapy was used to treat the child, and he had an excellent prognosis [8].

Although LCH is mainly a benign and curable condition, it can have long-term effects on the tissues involved. Some complications may be apparent at the time of diagnosis or even prior to it, while others may appear afterward. As a result, follow-up in these patients is critical at least until they have reached adulthood, if not longer. The most common long-term complications include endocrine and developmental problems, as well as auditory and orthopedic complications. Although neurocognitive, lung, and hepatic complications are uncommon, they produce significant morbidity [9]. LCH is an uncommon condition that manifests itself in a variety of ways. When an ear illness is resistant to medical treatment, a high index of suspicion is required to diagnose LCH. CT scan is tremendously helpful, but the diagnosis is dependent on the Langerhans cells being identified in biopsy specimens. There are several treatment methods available, all of which must be tailored to the patient's specific illness. Isolated lesions usually have a very good prognosis. In isolated lesions, complete surgical excision is successful. Follow-up is, however, essential and critical [10]. Similarly in our patient, CT scan of the mastoid was performed and the findings were helpful in the diagnosis of cholesteatoma, She was then successfully treated with mastoidectomy, with no event of recurrence.

## Conclusions

Cholesteatoma following LCH is a rare condition and is often difficult to diagnose due to its variable presentation, especially among children, and thus, requires a high index of suspicion for timely diagnosis and management. Secondary acquired cholesteatoma is usually present due to trauma or perforation. However, it can occur idiopathically, and surgical intervention is required. Therefore, surgery remains the recommended treatment, however, follow-up is essential in all cases.

## References

[1] Haupt R, Minkov M, Astigarraga I (2013). Langerhans cell histiocytosis (LCH): guidelines for diagnosis, clinical work-up, and treatment for patients till the age of 18 years. Pediatr Blood Cancer.

[2] Zheng H, Xia Z, Cao W, Feng Y, Chen S, Li YH, Wang DB (2018). Pediatric Langerhans cell histiocytosis of the temporal bone: clinical and imaging studies of 27 cases. World J Surg Oncol.

[3] Roger G, Dupré M, Leboulanger N (2009). Cholesteatoma secondary to temporal bone involvement by Langerhans cell histiocytosis: a complication amenable to curative surgery. Otol Neurotol.

[4] Simmonds JC, Vecchiotti M (2017). Cholesteatoma as a complication of Langerhans cell histiocytosis of the temporal bone: a nationwide cross-sectional analysis. Int J Pediatr Otorhinolaryngol.

[5] Dong-xiao N, Hui-tu N, An-zhou T, Ruo-ze C (2007). Langerhans' cell histiocytosis (histiocytosis X) of the temporal bone. J Otolog.

[6] Ardhaoui H HS, Abada R, Rouadi S (2019). Langerhans cell histiocytosis: manifestation in both mastoid cavities. Global J Otolaryngol.

[7] Ali MAQ, Al-Kindy SA (2017). Langerhans cell histiocytosis mimicking a complicated mastoiditis: a review and case report. Saudi J Health Sci.

[8] Sayore C, Bankole N, Rifi L, Cherradi N, Melhaoui A, Elouahabi A (2020). Spheno-temporal langerhans cell histiocytosis: a case report. Open J Modern Neurosurg.

[9] Donadieu J, Chalard F, Jeziorski E (2012). Medical management of langerhans cell histiocytosis from diagnosis to treatment. Expert Opin Pharmacother.

[10] ElSharkawy AA, Sarhan MM (2012). Langerhans' cell histiocytosis of temporal bone: a study of 11 Egyptian patients. J Int Adv Otol.

